# Extra-Limites

**Published:** 1840

**Authors:** 


### EXTRA-LIMITES.

### Observations on the Oxide of Silver; and an Abstract of the Cases in which it has been administered. By *C. H. B. Lane*, Esq.

The use of silver as a medicinal agent is of ancient date. By Avicenna it was given in the metallic form, as a remedy for palpitation of the heart and fœtid breath. Takennius used it combined with the cinnabar of antimony in melancholia, epilepsy, and mania. By Angelus Sala it was highly spoken of in the treatment of cerebral and uterine diseases. Mereat has recommended nitrate of silver strongly, as a remedy for hysteria and epilepsy, and relates a case of the latter of thirty years' standing, in which it effected a cure. It is not as much resorted to for internal administration as it was some years back, when its use was sanctioned by the names of Nord, Sprengel, Butini, Esquirol, Lombard, Baillie, Sims, and others. There are, however, still some advocates for its use in epilepsy and gastric affections, most especially Dr. James Johnson; and it has been given in dysentery and cholera. In 1836, M. Serre, of Montpellier, strongly recommended the various salts of silver, and also the oxide, as anti-syphilitics, but the evidence he advances of their utility does not appear satisfactory on his own showing, and M. Ricord's report on the subject is unfavourable.

The nitrate has been the form in which silver has been mostly employed; we owe it to alchymical research which, though futile as to its immediate object, has had the beneficial result of affording us numerous powerful weapons wherewith to combat disease. As a local application it is in very general use; it acts chemically as a caustic, and is supposed to possess peculiar stimulant powers in addition, which opinion was originally advanced by Mr. Higginbottom, whose work is, I believe, the only English monograph on the subject which we possess. In the present day there has been some tacit acknowledgment of a contra-stimulant influence, but it is vague and undefined. I do not believe nitrate of silver to possess any stimulant action beyond that connected with its causticity: any destructive process tends to excitement if reaction takes place, and will be indicated by pain, that vigilant sentinel of our corporeal welfare. The action of silver I consider as directly sedative as that of lead, only differently evinced. While the primary sedative action of lead is on the nerves of animal life, perhaps that of silver is in special relation to those of organic life, by which the capillary circulation is believed to be governed—this refers to the primary effect, for once the constitution affected, the sedative impression is conveyed to the nervous centres, and the further results may be varied. We will take a case of morbid vascular action occurring in a cutaneous surface, with a view to contrast the respective therapeutic action of lead and silver. We find the application of acetate of lead attended by diminution of sensation, and if the surface be at all abraded, some degree of pain may ensue from the contact of the foreign body, as also from the stimulation of the free acetic acid. The continued application of the lead may annihilate the sensation of the part, and that safeguard of the surface being destroyed, organic life is but too readily arrested, an evil to be carefully avoided. Suppose, on the other hand, we apply the nitrate of silver to what is termed an inflamed surface. It acts primarily as a caustic, becoming decomposed and combining with albumen, first as a chloride, and subsequently as an oxide, constituting a foreign body which protects the surface from external impressions, a mechanical effect well described by Higginbottom; and this process is attended with more or less pain in proportion to the liability of the surface to impression either from abrasion or excited sensibility: slight organic action even may ensue, causing serous effusion. The excitement, however, generally quickly disappears, and a distinct therapeutic action is evinced. The morbid state, whether demonstrated in increased capillary circulation, secretion, or absorbtion, subsides; when the affection is local it is often quickly subdued. In the incipient stage of anthrax there is no local application which equals nitrate of silver in efficacy; in several cases where freely applied, I have seen it arrest the local mischief which there was every reason to suppose would have otherwise gone through the tedious process of sloughing—suppuration and ulceration—and this was what induced me first to suspect that more than mere stimulation took place. Looking at the medicinal influence of nitrate of silver in erysipelas, and various forms of ulcer, in which the existence of excitement to a greater or less extent cannot be denied, I think we shall be still further inclined to lay aside the theory of mere stimulation.

The previous causticity of the lunar caustic is most advantageous in many cases, as for example, in the application to chancres and in chronic ophthalmia, where the destruction of morbid growth is desirable before there can be any susceptibility of sedative impression. There appears but little doubt that the application of nitrate of silver to syphilitic sores, if adopted while the affection is yet local, may preclude the necessity of constitutional remedies by destroying the centre of morbid action. Yet, on the other hand, the production of the sedative effect alone is often more advantageous, for where the organization is weak, the primary destructive causticity is badly borne, though, despite that inconvenience, nothing has been hitherto substituted which will as completely answer the purpose as lunar caustic carefully applied.

The question now is—can the caustic stimulation be avoided, and a simple sedative action be instituted through the agency of silver?—to this I would reply in the affirmative! I would recommend the substitution of the oxide for the nitrate, which stands in the same relation thereto as the oxides of antimony and mercury do to tartar emetic and corrosive sublimate respectively. Thus we shall have a mild and manageable preparation—entirely devoid of causticity, which my brief experience leads me to hope will be found of high utility both as a local and internal medicament.

I have applied an ointment of oxide of silver with decided effect in the various stages of gonorrhœa, and with but little inconvenience, beyond what might be fairly attributed to the mere mechanical irritation of the bougie. Over the nitrate it has the great advantage of not producing the caustic irritation by which, at the same time that the morbid secretion may be arrested by a process resembling tanning of the urethral lining membrane, there is considerable additional probability that metastasis of the irritation will ensue. The application of the oxide of silver, on the other hand, may be resorted to so as gradually to subdue the disease. I would not, however, by any means recommend its employment during the very acute stage while much constitutional irritation exists, and, subsequently, only as an auxiliary to the usual medicinal treatment. In chancres, I am convinced that I have seen the most beneficial results attend the application of the ointment. I have often found the sanative effect surprisingly accelerated after the failure of other remedies, and its use is not attended with pain. It is the oxide of silver which exists in Mr. Guthrie's black ointment, named by some "*infernal*" though far from violent in its action, and I believe available to a much greater extent than usually supposed. His formula consists of ten grains of nitrate of silver, fifteen drops of solution of lead, and a drachm of lard. The oxidization is effected by trituration and exposure to the atmosphere and light. A much readier mode of preparing it is from the oxide itself. The lead promotes sedative action but is not essential to it.

We will now turn to the internal administration of silver. The use of the nitrate has been strongly recommended by many in nervous diseases of all kinds, and also in gastric affections, which are especially states of excitement, though of course constitutional debility may exist, as is often the case. With the strong conviction of the powerful sedative influence of silver used externally, I could not doubt that the result of its internal administration would correspond from analogy with lead, of which the powerful influence over the nervous centres is indisputable. With respect to the nitrate, the risk of cutaneous discoloration has always been a great objection to persistence in its use, when it was deemed requisite for any lengthened period. Now the nitrate is a soluble salt, which is changed by the free hydrochloric acid of the gastric juice into a chloride: this is taken into the circulation, and when conveyed to the cutaneous surface is converted by its strong affinity for albumen and by the action of light into an oxide, which, however, cannot apparently permeate the capillaries, or we might expect it to be removed at a future period, as is the bile in jaundice. The oxide of silver, however, remains indelibly fixed; nor am I aware of any recorded instance of its perfect re-absorption. Again, the nitrate cannot be administered freely on account of its causticity, so that a full sedative impression is not obtained, perhaps even when the mischief of discoloration is done. I think I have fair reason to expect that the above evils would be avoided by substituting the oxide for the nitrate of silver. The transmutation of the salt in the cutaneous surface would then be anticipated, and its transmission thereto prevented; for as I just remarked, the cutaneous capillaries do not appear permeable to the oxide. We should, by freer administration, more readily impart the sedative impression through the medium of the nerves of the stomach, like that of other medicines of the same class. It was suggested to me that the oxide would probably be decomposed by the free acid of the gastric juice, but, from experiments I have made, I do not anticipate such a result. Experience alone can test the truth of the theory—to that I appeal, and by it am willing to abide. If it be successful, I shall have the satisfaction of adding to our store a therapeutic agent of peculiar utility.

The readiest mode of preparing the oxide of silver is by acting on the nitrate with potassa, we may remark that the grey nitrate of silver contains some portion of the oxide. The admixture of two drachms of the hydrate of potass with half an ounce of nitrate of silver will yield about three drachms of the substance in question. Its internal administration may generally commence in the dose of half a grain. I have not carried it beyond six grains in the twenty-four hours. I have used the ointment of oxide of silver with the proportion of from five to ten grains to the drachm of lard.

I will now briefly refer to the cases in which the remedy has been used within my own knowledge during the last twelve months. The unsuccessful cases are noticed in common with the successful ones, in order to afford some direction as to when the use is likely to be attended with advantage. A greater aggregate of success has been obtained than might have been anticipated from a mere experimental enquiry, which strongly encourages us to its continuance.

*Case* 1.—Mr. J. had two syphilitic sores on the glans penis which could not be got to heal during four or five weeks, though a variety of local applications were resorted to. With the use of the oxide of silver ointment they healed in ten days. At a subsequent attack he himself suggested the use of the ointment, which was used with the same satisfactory result. He did not take mercury.

*Case* 2.—Senor G. was labouring under exceedingly irritable chancre of phagadenic tendency. Black wash and ointment of the nitric oxide of mercury were ordered for him by Mr. Guthrie, who saw him with me in consultation. At the end of a fortnight he was worse rather than better. I then used the oxide of silver ointment, which effected the healing process in a week.

*Case* 3.—Mr. M. had syphilitic sores on the prepuce. Caustic was applied in the first instance, and subsequently the oxide of silver ointment daily. The chancres were well within a week, but he subsequently suffered severely with secondary symptoms.

In many other cases of syphilis I have since been constantly in the habit of using the ointment in question, and have generally found it answer better than any other local application. Also other medical men, who have been induced to try it by my recommendation, have expressed themselves much pleased with its efficacy.

*Case* 4.—Gleet and stricture. The application of the ointment with a bougie produced temporary benefit only— it occasioned no pain.

*Case* 5.—Mr. W. a gentleman of plethoric habit had the ointment applied a fortnight from the first commencement of gonorrhœa. No pain was experienced at the time, but there was much subsequent irritation.

*Case* 6.—F. J. a newly-married man, laboured under severe gonorrhœal symptoms, apparently caused by leucorrhœa in his wife. He was at intervals under treatment with the local application of oxide of silver during a period of five weeks, always with marked relief, but the complaint kept recurring till he became insensible to the exciting cause. He was a patient of Mr. P. B. Lucas.

*Case* 7.—S. L. had been affected with gonorrhœa some days, when the ointment was first applied. The appearance of glandular irritation in the groin precluded its continuance.

*Case* 8.—J. M. became affected with purulent discharge, ardor urinæ, and chordee, four days after connexion. He was ordered an aperient, and the ointment was applied three successive days, when he reported himself well. He was a patient of Mr. P. B. Lucas.

From these and other cases of the kind, in which the ointment was used without resorting to other remedies at the same time, I should not feel induced to rely on it solely in cases of urethral discharge, but am satisfied that it will often be found an useful adjunct in cases of that description.

*Cases* 9 and 10.—Extensive ulcers of the legs of old-standing were benefitted to a slight extent, but the use of the ointment was not persisted in.

*Case* 11.—Rachel G——, æt. 32, of nervo-bilious temperament. Has suffered much from mental distress, which has been accompanied with gradual deterioration of her bodily health. Pulse quick and weak—tongue rather dry—and menstruation irregular, often in excess. It has now continued ten days, and there is also diarrhœa, the bowels acting as soon as food is taken into the stomach. There is constant pain in the head and epigastrium—leucorrhœa—globus hystericus—and anorexia. She was ordered a little mercurial pill with extract of conium each night, and half a grain of oxide of silver twice a day. On the next day of seeing her there was slight general improvement. The diarrhœa and menorrhagia were restrained. She complained of a sensation of pain and weight in the stomach after taking the pills. Symptoms of febrile catarrh subsequently occurred, and she complained of the pills occasioning pain and making her sick—they were, therefore, after ten days, discontinued. In as much as the state of mind and a very sedentary occupation were apparently the permanent and essential causes of the woman's complaint, removing it out of the sphere of medicinal relief, the hope of material amelioration by any remedy appeared precluded. She was a patient of Dr. Golding Bird, at the Finsbury Dispensary.

*Case* 12.—M. P. æt. 50. had suffered ever since the cessation of the menses, at the age of 45, with gastric and uterine irritation, indicated by frequent vomiting, loathing of food—flatulency—sour eructations—constipation—pain in the back—sense of bearing down—and leucorrhœa. She was in a state of excessive debility. Had been under the care of Sir James Clarke, and others, without deriving any benefit. In the course of a fortnight, with the use of mild aperients and half-grain doses of oxide of silver twice and three times a day, she was greatly relieved, and from that time rapidly progressed to recovery. She was under the care of my friend Mr. C. J. Phillbrick, of Colchester.

*Case* 13.—Sarah K. æt. 54, was complaining of a dull aching pain occurring in the epigastrium, chiefly after eating, together with a sense of fulness. Pulse quick and rather sharp. Appetite bad—bowels regular. The tongue anteriorly presents a strawberry and cream appearance, posteriorly there is thick white fur ceasing abruptly. She was ordered an aperient and half-grain doses of oxide of silver twice a day. The pills at first made her feel sick, and occasioned a sense of gastric constriction; but this soon went off, and she felt altogether better. The amelioration continued for a time, when constant sickness supervened. The remedy was then discontinued, and I am inclined to consider the case as one where disorganizing action was going on in the stomach.

*Case* 14.—J. D. was first seen by me September 3d, 1839. He had been suffering with gastralgia during five months, and had derived but slight benefit from stimulants, opiates, or counter-irritation. He complained of constant pain in the scrobiculus cordis, violently exacerbated once or twice daily, the paroxysm sometimes lasting two hours, and of such intensity as to compel him to lay down wherever he might be. No sickness, pulse natural, bowels regular. A sensation of emptiness was experienced before eating, and soreness and fulness afterwards. He was ordered half a grain of the oxide of silver three times a day. On the 5th he expressed himself much relieved: there was less tenderness, and he felt as if something was removed from the stomach: the unpleasant sensation before and after eating was also much diminished. He went on favorably till the 19th, when the symptoms recurred, but were again subdued by the administration of the remedy in doses of a grain and a half three times a day. The man was a patient of Dr. Ryan.

*Case* 15.—Mrs. M. æt. 37, was affected with complicated and anomalous abdominal disease, of which, suffice to say, diarrhœa and pyrosis constituted distressing symptoms, which were relieved in forty-eight hours by the use of oxide of silver. The pyrosis had existed previously for three months. She was seen twice by Dr. Todd.

*Case* 16.—Mr. L. æt. 44. had been for some years on and off affected with pyrosis, gnawing pain in the stomach, and various other dyspeptic symptoms, which all ordinary means failed in alleviating. Mr. Dennett, of Storrington, Sussex, whose patient he was, reported him completely relieved with the oxide of silver.

*Case* 17.—T. D. had suffered at times for the last five years with severe gastralgia, generally occurring towards Autumn. There was constant aching and tenderness in the region of the stomach. He also complained of nausea, and at times a quantity of clear water was thrown up. Anodynes, bleeding, calomel and opium, stomachics, bismuth, and counter-irritation were in turn resorted to without benefit. After three months, oxide of silver was prescribed, and from it complete relief was speedily obtained. Dr. Todd saw this patient two or three times.

*Case* 18.—Jane W. æt. 25, had been affected with occasional pain of a gnawing character in the left hypochondrium for the last three years: it sometimes came on very violently, but without reference to the stomach being full or empty, and has been much worse for the last *few* weeks. Bowels regular, and general health pretty good, but "the pain wears her out." At the expiry of three weeks complete relief was afforded by administering the oxide of silver in half-grain doses twice daily, and she had become much stronger and more cheerful. She was a patient of Dr. Golding Bird, at the Finsbury Dispensary.

*Cases* 19, 20, and 21.—Oxide of silver was administered to three women suffering with leucorrhœa, during periods varying from a week to a fortnight, but no essential benefit accrued. The discharge was merely vaginal.

*Case* 22.—Jane C. æt. 25, unmarried, of a sallow and aged appearance, complained of great general languor and debility, with loss of appetite. She had been affected eleven years, ever since the commencement of menstruation, with uterine irritation, and apparently in connexion therewith by fits of an epileptic character, but she does not usually scream. They generally come on in the night time, and have latterly been much more frequent, occurring once a fortnight, immediately after the menstrual period and between whiles: they were preceded by swelling of the genitals. Under the use of the pills she improved greatly in the first instance, one menstrual period passing without fits. The improvement continued for a fortnight, but the temporary lull was only fallacious, the fits coming on as badly as ever. She was a patient of Dr. Golding Bird, who deemed the case one of epileptic hysteria.

*Case* 23.—Esther J. æt. 16, evidently of precocious bodily development. She had the appearance of a woman of 25, and had evidently outgrown her strength, so that it was a question whether spinal irritation might not exist. The menses had appeared two years previously, but since been somewhat irregular. Countenance pallid—bowels torpid—occasional sickness—pain in the left hypochondrium—pulse 80. She was ordered aperients and half a grain of oxide of silver twice a day. Instead, however, of getting better, she got worse—the pain and sickness increasing, and the pulse being accelerated. The remedy was therefore discontinued.

*Case* 24.—Sarah B. æt. 17, complained of severe pain in the left side, her health being otherwise excellent. The menstrual function had been decayed, but was re-established by the use of electricity. She was ordered half-grain doses of oxide of silver, which at first made her feel somewhat sick, but by persisting in its use for near a fortnight the complaint was effectually relieved. She was a patient of Dr. Golding Bird, as were also the next four persons to whose cases I am about to refer.

*Case* 25.—Mary H. æt. 31, suffered severely with pain in the left side, and was also somewhat hysterical and weakly. A fortnight's administration of the oxide of silver completely removed the pain, and effected much improvement in her general health.

*Case* 26.—Marianne P. æt. 21, had suffered severely for some months with pain in the left side, which was constant, but becoming much worse at times. Was troubled with cough and sickness, though otherwise in good health. A fortnight's course of oxide of silver completely relieved the lateral pain.

*Case* 27.—George V. æt. 45, of bilious temperament and sedentary occupation, was suffering with severe burning pain under the right scapula, occasional sickness, severe cough, loss of appetite, diarrhœa, (the bowels acting eight or ten times a day,) and sense of fulness about the abdomen: there was much cardiac and vascular excitement, and he suffered from general debility. Half a grain of oxide of silver was ordered twice a day. The diarrhœa was soon restrained (the bowels, indeed, becoming somewhat constipated), the stomach was more settled and the scapular pain abated. He continued the pills with the addition of a small quantity of decoction of aloes and compound camphor tincture, so as to allay the cough and regulate the bowels. Under the above treatment the case progressed most favorably, and at the expiry of three weeks, he was perfectly relieved in every respect, excepting his cough, feeling stronger and more comfortable than he had done for a long time.

*Case* 28.—Sarah S. æt. 33, had suffered with hæmorrhage from the bowels, ever since her confinement nine weeks previously, by which she had become much reduced. She had been under treatment with lead, and for some time without any permanent effect, constantly losing much blood of a venous character, especially when the bowels acted, which they did regularly twice a day. No piles could be detected. The skin was dry—the pulse weak—the face œdematous, and the lacteal secretion nearly suppressed. She took the oxide of silver first in half-grain, and subsequently in grain doses, every six hours, and within sixty hours the hæmorrhage was effectually arrested: the medicine was continued for about a fortnight at longer intervals, and a rapid re-establishment of her health was effected.

*Case* 29.—Mrs. F. had been affected for ten days with profuse menorrhagia, the blood being partly discharged in clots, and, according to her account, in very great quantity. The bowels were open—the pulse was weak and quick. Dilute sulphuric acid, opium, and acetate of lead, in turn, failed to restrain the flux. The last remedy appeared to take some effect, but was compelled to be laid on one side, from disagreeing violently with the stomach. A strong infusion of secale cornutum was then administered, but caused violent vomiting without having any apparent effect on the hæmorrhage. Half-grain doses of the oxide of silver were then combined with the infusion of the ergot, when the vomiting no longer took place, and the menorrhagia was arrested within eight and forty hours.

*Case* 30.—G. D. æt. 27, had suffered with epilepsy from his boyhood, for which he had submitted to much medical treatment. In August last he had been taking nitrate of silver for nearly two months, under Dr. Burne's directions, without benefit. For two months I then administered the oxide at the rate of from a grain and a half to five grains per diem. It was then laid aside for a fortnight, in consequence of a deranged state of bowels. He then resumed it for a period of six weeks, when he desisted in consequence of *apparent salivation*, which was supposed at the time to depend on alveolar abscess, situated at the root of one of the molar teeth. He resumed the medicine after a short time at the rate of four grains per diem, but in the course of ten days was again salivated, evidently from its use, and while in this state he was seen by Dr. James Johnson. The further use of the remedy was therefore precluded, but an instance was constituted of the constitution being powerfully under the medicinal influence of the oxide of silver from a lengthened administration without cutaneous discoloration resulting. The fits became on the average much less frequent and severe at the time, and he enjoyed a much better state of general health, being stronger and less nervous than previously. The man has, I regret to say, since relapsed, and his fits are as bad as ever. In another epileptic case oxide of silver was administered under the direction of Dr. James Johnson, for the space of two months, without any inconvenience resulting, but, not appearing to exert a beneficial influence over the disease, it was laid aside.

In relation to the above cases the following brief considerations may be advanced. The oxide of silver is entirely devoid of causticity, its local application occasioning no pain, a valuable fact in reference to its internal administration. The remedy appears beneficial in various nervous affections, when they have become idiopathic, that is to say, when the cause, whether originally seated in the stomach, uterus, spinal cord, or other viscus, is removed, and the impression alone remains behind. There are no cases in which the oxide of silver is so rapidly beneficial as in cases of idiopathic gastric irritation, whether evinced in pyrosis, gastrodynia, or want of relation between the stimulus of food and the action of the stomach; but if organic change have taken place in the organ, the same benefit is not to be anticipated. In obstinate diarrhœa and hæmorrhages I am greatly in hopes that the silver will be found analogous in its action to lead—as efficacious, but milder and more manageable in its effect; this however requires much further trial. It would be unfair that the merits of the oxide of silver should be at all suffered to rest on its efficacy in epilepsy, of which we are well assured that the great majority of cases depend on organic change, which the medicine cannot influence. In reference to its utility in epileptic cases, all that can be said is, that if, as I consider, the oxide of silver will not produce a discolouring effect on the skin, the utmost advantage that can be expected from the dangerous administration of the nitrate may be safely and fearlessly attained by the substitution of the oxide.

I shall conclude by adducing the evidence on the subject which has been kindly afforded me in written communications.

Dr. Clendinning informs me that—

"The oxide of silver has, so far as tried at the Marylebone Infirmary, appeared to exert an influence in epileptic and gastralgic affections similar to that long attributed to the nitrate of silver. The particulars of two or three cases in which the medicine appeared to act decidedly beneficially, have been communicated to Dr. C. by the resident officers, and other cases have been mentioned to him by the same gentlemen in which more or less relief seemed to attend the use of the remedy."

Dr. Ryan writes thus—

"I have tried the oxide of silver in very bad cases of epilepsy, I think with some benefit." "From these I am disposed to think that it may prove a valuable remedy, but my experience is at present too limited to justify a positive conclusion. It had a very decided effect in intense gastralgia."

Dr. Golding Bird makes the following observations .—

"From the experience I have had in the administration of the oxide of silver, I have formed a high opinion of its value as a therapeutic agent; not from its possessing any marvellous specific power, but from its tonic, and to a certain extent, sedative properties—rendering it as far as I have seen an useful remedy in several forms of neuralgia, and especially in certain cases of dyspepsia attended with irritable stomach and pain in that viscus after taking food, when the secretions of the liver and intestines have been corrected as much as possible by the careful administration of alteratives. The oxide of silver appears to me to possess the good qualities of the nitrate without its inconveniences, and to exert a more directly sedative action than that salt; in which property indeed it approaches the acetate of lead, at least if the effects of its administration in menorrhagia can be admitted as sufficient grounds to justify this conclusion."

